# Supplementary material for: Functional Analysis of a Novel, Non-Canonical RPGR Splice Variant Causing X-Linked Retinitis Pigmentosa
Source: Genes (Basel). 2023 Apr 18;14(4):934. doi: 10.3390/genes14040934 (PMC10137330; doi:10.3390/genes14040934)
Supplement: Supplementary file 1 [file genes-14-00934-s001.zip › Figure S2 Coverage of ORF15 in WES data.pdf]

S2

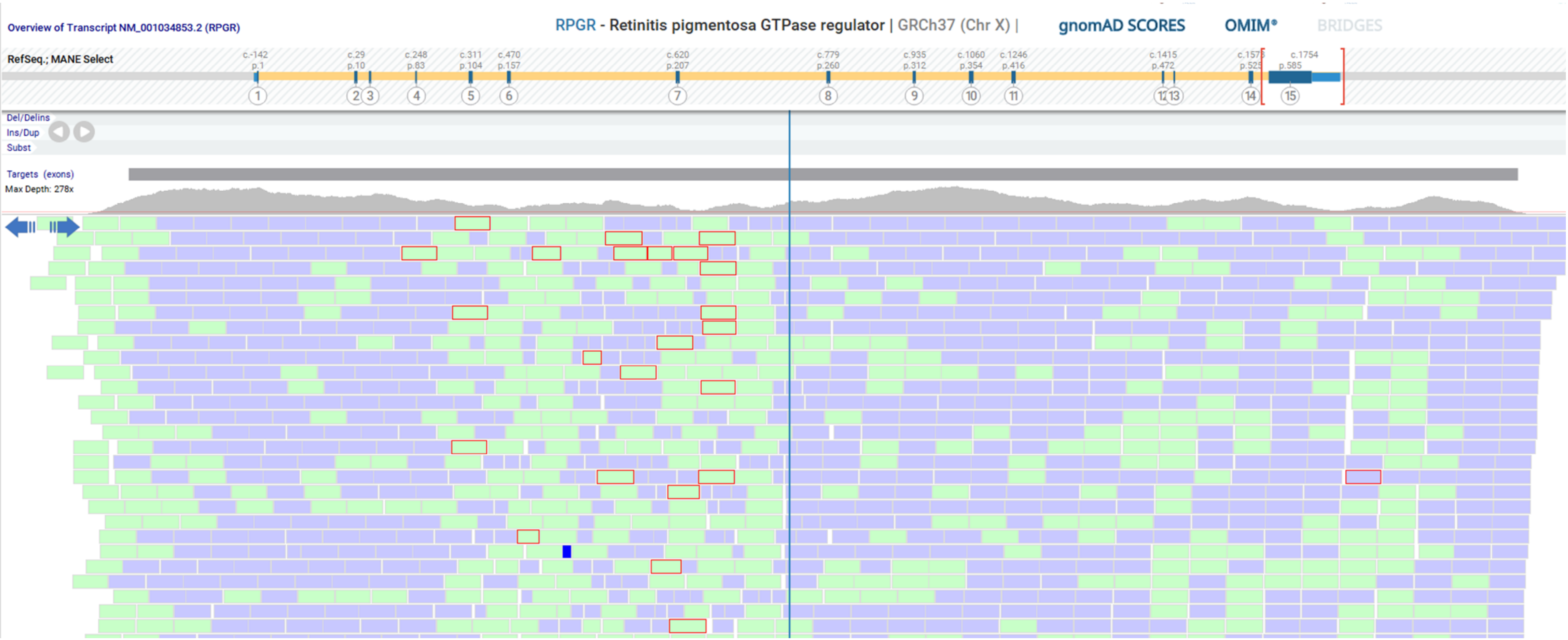
Overview of Transcript NM_001034853.2 (RPGR)
RPGR - Retinitis pigmentosa GTPase regulator | GRCh37 (Chr X) |
gnomAD SCORES
OMIM®
BRIDGES
RefSeq.; MANE Select
Del/Delins
Ins/Dup
Subst
Targets (exons)
Max Depth: 278x
