# Supplementary material for: Functional Analysis of a Novel, Non-Canonical RPGR Splice Variant Causing X-Linked Retinitis Pigmentosa
Source: Genes (Basel). 2023 Apr 18;14(4):934. doi: 10.3390/genes14040934 (PMC10137330; doi:10.3390/genes14040934)
Supplement: Supplementary file 1 [file genes-14-00934-s001.zip › supplemental methods and figure legends.pdf]

SUPPLEMENTAL MATERIAL AND METHODS

Molecular cloning

Primers:

RPGR_ex12_RHO_for:

CCCTGGAGGAG *CCATGGTCTGG* <u>CTGTGCACATACGGACCA</u>

Gene specific Tm: 59.5 (<u>underlined</u>)

Restriction site: PflMI (*italic*)

RPGR_ex12_RHO_rev:

TCG GAG GTA *CCTCTCCGAGG* <u>AAATGAAGGGAAAACAGTTCAGA</u>

Gene specific Tm: 58.6 (<u>underlined</u>)

Restriction site: EcoNI (*italic*)

PCR program Phusion-polymerase:

1. 98°C 30s
2. 98°C 10s
3. 62°C 20s
4. 72°C 15s repeat steps 2-4 35x
5. 72°C 10min
6. 10°C until end

Transcript analysis:

Primers 60°C Tm:

T7 for:

TAATACGACTCACTATAGG

BGH rev

TAGAAGGCACAGTCGAGG

RPGR_exon11_for:

TCTTGGCCTTTCTGCTTGTT

RPGR_exon13_rev:

TTCATTGGAATTCAGGCTCA

PCR Program HOT FIREPol (Taq) polymerase

1. 15 min 95°C
2. 1 min 95°C

3. 1 min 60°C
4. 2 min 72°C repeat steps 2-4 35x
5. 10 min 72°C
6. 10°C until end

Supplemental Figure S1

A) Sequencing analysis of the index by NGS of *RPGR* shows the variant in a hemizygous state. Sanger analysis confirmed heterozygous state of that variant in the mother.

B) PCR analysis of minigene transcript of RHO-RPGR-RHO hybrid gene construct. T7 and BGH primers were used to amplify the transcript generated from the plasmid in HEK293 cells. The expected size of the fragment is 516 bp. smaller fragment represents *RPGR* exon 12 skipping (*RPGR* exon 12 size is 92 bp).

C) PCR analysis of *RPGR* cDNA derived from blood of the index and its mother. Primers bind to exon 11 and 13 of *RPGR* were used for PCR. Expected size of the fragment is 235 bp. Weak smaller fragment represents *RPGR* exon 12 skipping.

Supplemental Figure S2

Overview of whole exome sequencing coverage for *RPGR* ORF15
