# Supplementary figures and images for: Functional Analysis of a Novel, Non-Canonical RPGR Splice Variant Causing X-Linked Retinitis Pigmentosa
Source: Genes (Basel). 2023 Apr 18;14(4):934. doi: 10.3390/genes14040934 (PMC10137330; doi:10.3390/genes14040934)

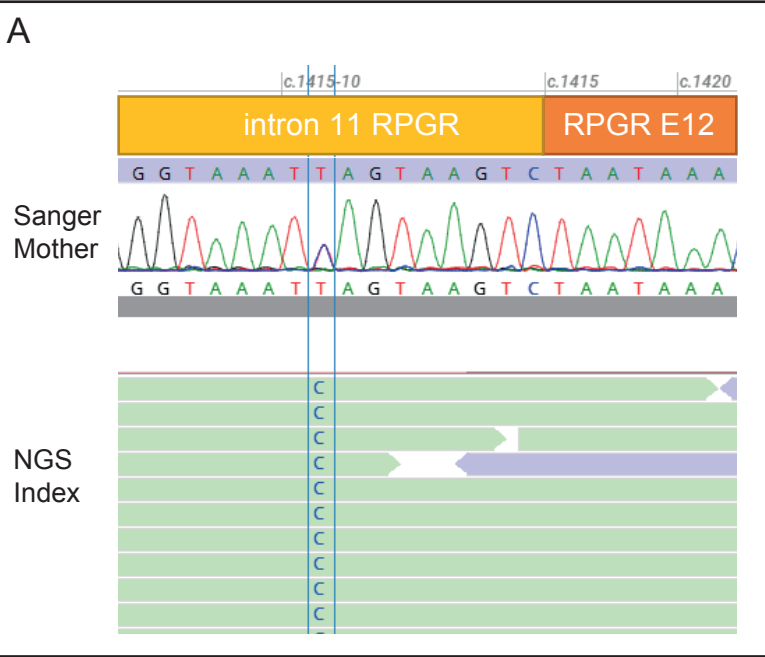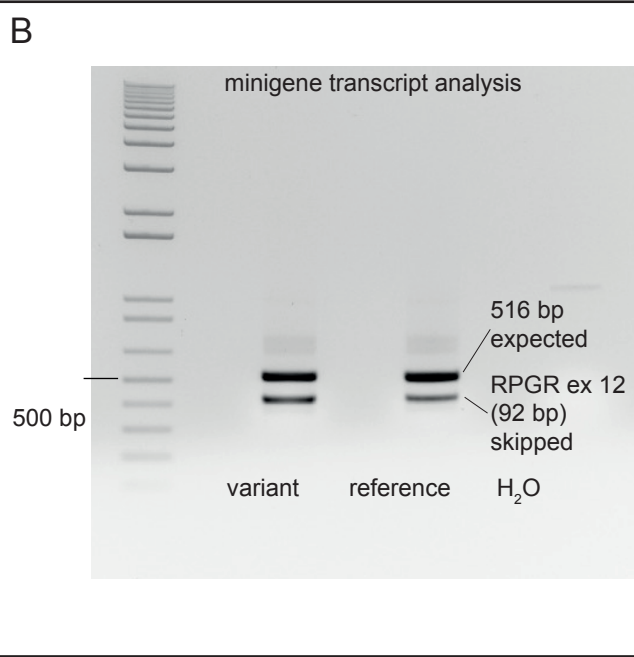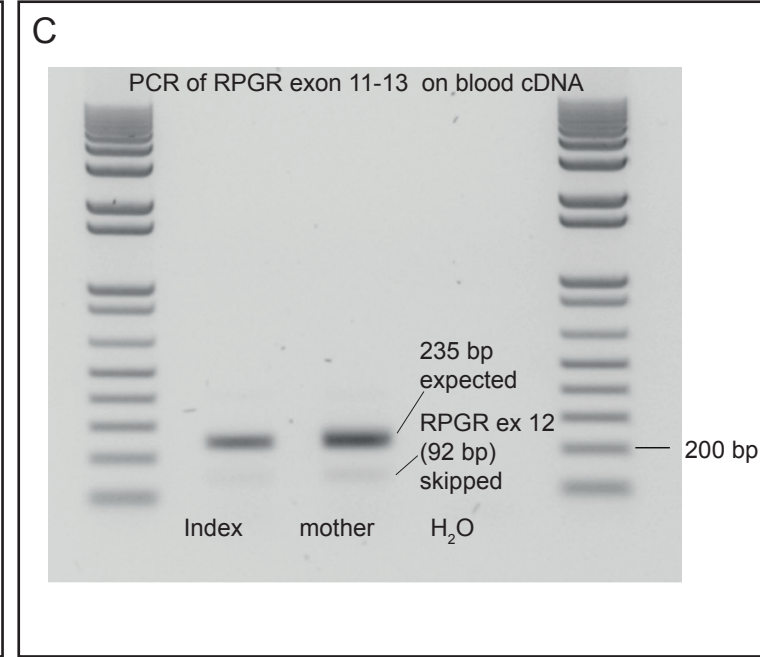

Supplement: Supplementary file 1 [file genes-14-00934-s001.zip › Figure S1 Sequencing data and PCR analysis of transcripts.pdf]
